# Evaluation of Fatty Acid and Amino Acid Compositions in Okra (*Abelmoschus esculentus*) Grown in Different Geographical Locations

**DOI:** 10.1155/2013/574283

**Published:** 2013-09-22

**Authors:** Rokayya Sami, Jiang Lianzhou, Li Yang, Ying Ma, Jing Jing

**Affiliations:** ^1^Department of Food Science, Northeast Agricultural University, Harbin, Heilongjiang 150030, China; ^2^Department of Home Economics, Faculty of Education Quality, Mansoura University, Mansoura, Dakahlia 35516, Egypt; ^3^School of Food Science and Engineering, Harbin Institute of Technology, Harbin, Heilongjiang 150090, China

## Abstract

Okra has different uses as a food and a remedy in traditional medicine. Since it produces many seeds, distribution of the plant is also quite easy. Although seed oil yield is low (4.7%), since the linoleic acid composition of the seed oil is quiet high (67.5%), it can still be used as a source of (UNSAT) unsaturated fatty acids. In this study, samples of okra grown in four different locations were analyzed to measure fatty acid and amino acid compositions. The content of the lipid extraction ranged from 4.34% to 4.52% on a dry weight basis. Quantitatively, the main okra fatty acids were palmitic acid (29.18–43.26%), linoleic acid (32.22–43.07%), linolenic acid (6.79–12.34%), stearic acid (6.36–7.73%), oleic acid (4.31–6.98%), arachidic acid (ND–3.48%), margaric acid (1.44–2.16%), pentadecylic acid (0.63–0.92%), and myristic acid (0.21–0.49%). Aspartic acid, proline, and glutamic acids were the main amino acids in okra pods, while cysteine and tyrosine were the minor amino acids. Statistical methods revealed how the fatty acid and amino acid contents in okra may be affected by the sampling location.

## 1. Introduction

 Okra (*Abelmoschus esculentus*) is widely distributed in tropical to subtropical regions in Africa, Asia, Southern Europe, Mediterranean countries, and America. Okra is mainly grown as a vegetable in the plains of Egypt. It grows well under warm climatic conditions (temperatures above 26°C). 

 The seeds of mature okra pods are sometimes used for poultry feeding and are also consumed after roasting and as a coffee substitute. They are considered to be a stomachic stimulant, antispasmodic, and nervine [1]. Okra seeds have been used on a small scale for oil production. Okra seeds from Greece are a potential source of oil, with concentrations varying from 15.9% to 20.7% [2]. The oil mainly consists of linoleic acid (up to 47.4%) [3]. Okra seed oil is a rich source of unsaturated fatty acids. The use of natural components in reducing cardiovascular diseases, cerebrovascular diseases, and cancer mortality has gained considerable attention. Lipid components greatly contribute to the nutritional and sensory value of almost all types of foods. Nature provides a large number of fats that differ in their chemical and functional properties. Four classes of lipids are habitually found in vegetable oils: triacylglycerols, diacylglycerols, polar lipids, and free fatty acids. The fatty acid composition determines the physical properties, stability, and nutritional value of lipids.

The most naturally occurring storage lipids are triacylglycerols. Triacylglycerols are natural compounds that consist of saturated and unsaturated fatty acids that differ in the length of their acyl chains and the number and positions of double bonds: saturated, monoenoic, and polyunsaturated fatty acids that differ with respect to detailed fatty acid composition. Monoenoic fatty acids and polyunsaturated fatty acids are structurally distinguished by the presence of repeating methylene units. These units produce an extremely flexible chain that rapidly reorients through conformational states and constitutes an influential group of molecules that promote health [4]. Proteins play a particularly important role in human nutrition. The amino acid contents, proportions, and their digestibility by humans characterize a protein's biological value [5]. Okra has been called “a perfect villager's vegetable” because of its robust nature, dietary fiber, and distinct seed protein balance of both lysine and tryptophan amino acids (unlike the proteins of cereals and pulses) [6, 7]. The essential and nonessential amino acids in okra are comparable to those in soybeans. Hence, it plays a vital role in the human diet [8]. 

 The aim of the present study was to investigate and compare total lipid, fatty acid, and amino acid composition in okra. In addition, the study was designed to obtain a comprehensive and detailed profile of the different components of okra pods, which may be of both industrial and nutritional interests.

## 2. Materials and Methods

### 2.1. Plant Material

 Okra pods were collected from different locations in Egypt: S pod (Suez) near the desert, M pod (Mansoura) near the Nile River, K pod (Kafr El-Sheikh) near the Mediterranean sea, and D pod (Dakahlia) near a lake. The pods were sun-dried, and their contents were analyzed.  Table 1 presents more information about the geographical origins and lipid contents.

### 2.2. Soxhlet Extraction of Lipids

 Lipids were extracted using the method of Soxhlet [9]. All solvents were of reagent grade and purchased from Sigma Chemical Co. (St Louis, MO, USA) and were used without any further purification. About 15 g of okra was ground in a coffee mill and immediately extracted, in duplicate, with 200 mL of hexane and heated at 35–60°C for 6 h at the rate of 2-3 drops/s. Hexane was removed using a rotary evaporator at 40°C in a vacuum, and the extracts were dried to a constant weight; then, the residue was stored at −20°C in the dark for fatty acid analysis [10, 11]. 

### 2.3. Fatty Acid Measurement 

*Fatty Acid Methyl Ester Preparation*. Fatty acid methyl esters (FAMEs) were prepared according to [12]. An aliquot (1 mL) of total lipids was evaporated in a tube of methylation. Fatty acids were saponified with 10 mL of methanolic sodium hydroxide solution (0.5 M) for 15 min in a boiling water bath at 65°C. For transmethylation, the mixture was homogenized with 10 mL of methanolic solution of BF_3_ (20%, w/v), and the reaction was allowed to proceed for 5 min. FAMEs were extracted twice with 10 mL of petroleum ether and 10 mL of water being added to the mixture.

### 2.4. Gas Chromatography and Gas Chromatography-Mass Spectrometry Analyses

 FAMEs were analyzed using gas-liquid chromatography (model HP 6890; Agilent, Palo Alto, CA, USA) equipped with a flame ionization detector. An SP-2560 fused silica capillary column (i.d., 100 m × 0.25 mm; film thickness, 0.2 *μ*m; Supelco, Inc., Bellefonte, PA, USA) was used. The column parameters were as follows: initial column temperature was held at 40°C for 5 min after injection, 20°C/min to 220°C, and finally the temperature was held at 220°C for 30 min. Helium was the carrier gas, with the column inlet pressure set at 17 psi. The detector temperature was 250°C. For identification purposes, these analyses were also performed with a gas chromatograph (model HP 6890; Agilent) coupled with a 5973 mass spectrometer detector (Agilent) by using the same column described previously.

 FAMEs were identified by using standards (Supelco 37 Component FAME mix; Supelco Bellefonte, PA, USA) and comparing their mass-spectral data with the mass-spectral database in the Wiley 7.0 library (HPMass Spectral Libraries, Palo Alto, CA, USA). The conjugated linoleic acid peaks were identified by comparison with the retention times of the reference standard (conjugated linoleic acid methyl ester, a mixture of *cis*-9, *trans*-11 octadecadienoic acid methyl ester and *cis*-10, *trans*-12 octadecadienoic acid methyl ester; Sigma Chemical Co.). Fatty acid contents were expressed as the proportion of each individual fatty acid to the total amount of all fatty acids present in the sample.

### 2.5. Amino Acid Measurement

 Sample aliquots containing around 8–12 mg of proteins were placed in a 20-mL cuvette and mixed with 9 mL of 6 M HCl [13]. After sealing the cuvette, the samples were hydrolyzed at 110°C for 24 h under N^2^. The hydrolysates were transferred into a 100 mL volumetric flask, mixed with 9 mL of 6 M NaOH, and diluted with 0.02 N HCl. Then, all the samples were filtered and loaded in a Hitachi L-8800 amino acid analyzer (Tokyo, Japan) for amino acid analysis.

### 2.6. Statistical Analysis

 Data from the replications of all varieties were subjected to a variance analysis (ANOVA) using SPSS 16.0 for Windows. Significant differences between the means were determined by Duncan's new multiple range test (*P* < 0.05). The correlation between all the studied parameters was determined by the principal component analysis (PCA) using XLSTAT software. 

## 3. Results and Discussion

### 3.1. Results of the Fatty Acid Analysis

**Figure 1:**
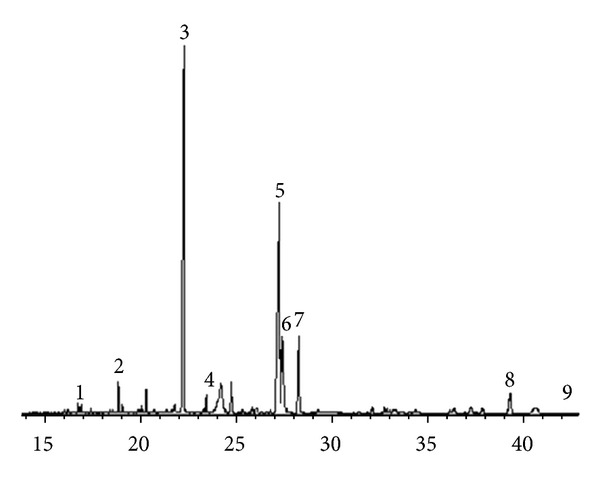
Typical chromatogram of fatty acid methyl ester prepared from K pod variety oil. Peaks: 1, Myristic acid; 2, Pentadecylic acid; 3, Palmitic acid; 4, Margaric acid; 5, Linoleic acid; 6, Oleic acid; 7, Linolenic acid; 8, Stearic acid; 9, Arachidic acid.

 The fatty acid composition of the lipids extracted from sun-dried okra plants is presented in Table 2. The okra plants had a low amount of oils. The lipid content did not vary significantly among okra pods; it ranged from 4.34 g/100 g for M pods to 4.52 g/100 g for S pods. All the studied okra pods had higher fat content than the values previously reported for okra [14]. An examination of FAME derivatives showed nine fatty acids. The total saturated fatty acids (SFA), monounsaturated fatty acids (MUFA), and polyunsaturated fatty acids (PUFA) showed significant variation in their contents. Palmitic acid (29.18–43.26%) was the major fatty acid; it promotes natural oil regeneration. Oil is an important component for the skin to retain its protective barrier. With too little oil, the skin will crack and bleed, resulting in a greater risk of infection and disease. The next most common fatty acid was linoleic acid (32.22–43.07%), which was most abundant in the S pod, followed by linolenic acid (6.79–12.34%), stearic acid (6.36–7.73%), oleic acid (4.31–6.98%), arachidic acid (ND–3.48%), margaric acid (1.44–2.16%), pentadecylic acid (0.63–0.92%), and myristic acid (0.21–0.49%).  Figure 1 shows chromatograms of a fatty acid sample. In all the cases, saturated fatty acids (SAT) predominated over SFA, ranging from 67% to 117%, and particularly, PUFA predominated over MUFA. Nine fatty acids were identified and quantified. To the best of our knowledge, there are no previous reports on the fatty acid composition of okra pods. The present study proved that okra pods are a source of beneficial fatty acids such as the polyunsaturated fatty acids linoleic and *α*-linolenic acid. Linoleic acid is a member of the group of essential fatty acids called omega-6 fatty acids, so called because they are an essential dietary requirement for all mammals and promote the biosynthesis of arachidonic acid, and thus, some prostaglandins. Linoleic acid is used in making soaps, emulsifiers, and quick-drying oils. It has become increasingly popular in the cosmetics industry because of its beneficial properties on the skin, including anti-inflammatory, acne-reduction, and moisture-retention properties [15]. Studies have found evidence that *α*-linolenic acid, a polyunsaturated omega-3 fatty acid, is related to a lower risk of cardiovascular disease [16]. Prostaglandins and thromboxanes are related compounds known as eicosanoids, which have a large variety of biological activities, including mediation in anti-inflammatory processes, lowering of blood pressure, relaxation of coronary arteries, and inhibition of platelet aggregation [17]. 

### 3.2. Results of the Amino Acid Analysis

**Figure 2:**
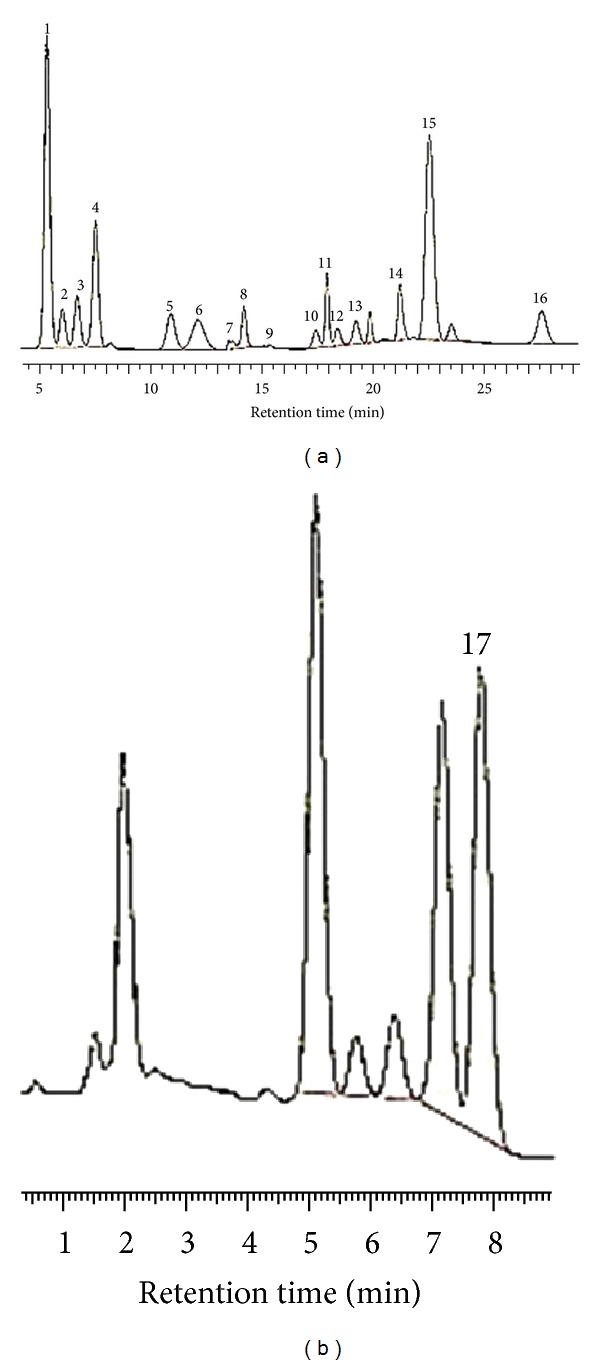
Typical chromatogram of amino acid from K pod variety. Peaks: 1, arginine; 2, threonine; 3, serine; 4, glutamic acid; 5, glycine; 6, alanine; 7, cysteine; 8, valine; 9, methionine; 10, isoleucine; 11, leucine; 12, tyrosine; 13, phenylalanine; 14, lysine; 15, histidine; 16, Linolenic acid; 17, proline.

 The amino acid profile of the okra plants is shown in Table 3, listing the concentrations of 17 amino acids. Among these amino acids, 11 essential amino acids were found. The major amino acids were aspartic acid (2.91–4.92 g/100 g), followed by proline, glutamic acid, arginine, leucine, alanine, lysine, serine, and phenylalanine. Methionine, isoleucine, histidine, cysteine, and tyrosine were the minor amino acids in okra pods. The major acids constituted more than 76.45% of the total amino acids present in the proteins of the okra plants. The total amount of nonessential amino acids (*N*) was higher than that of the essential amino acids (*E*). M pod and K pod were found to be rich in isoleucine, lysine, and valine, with a combined concentration of 1.32 g/100 g for M pod and 1.46 g/100 g for K pod. However, significant differences (*P* < 0.05) in arginine, aspartic acid, and proline contents were observed between M pod and K pod. The amount of sulfur-containing amino acids (methionine and cystine) was 0.24, 0.23, 0.30, and 0.19 g/100 g for S, K, M, and D pod, respectively, while the total aromatic amino acid content was 0.66–0.96 g/100 g. D pod showed the lowest value, and M pod showed the highest value.  Figure 2 shows chromatograms of the amino acid samples.

### 3.3. Results of the Principal Component Analysis

**Figure 3:**
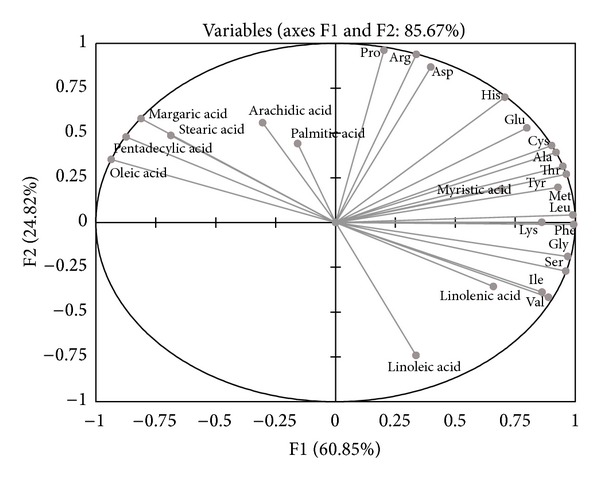
Plots of the scores for fatty and amino acids content of okra pods.

**Figure 4:**
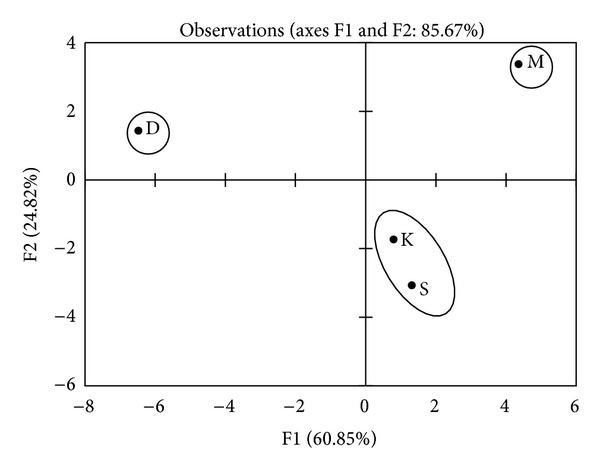
Plots of the *x*-loudings for fatty and amino acids content of okra pods.

 PCA was used to analyze the fatty acid and amino acid contents. Figures 3 and 4 present the plots of the scores and the correlation loadings, respectively. The scores plot of PCA illustrates the large variability of the four okra varieties (S, M, K, and D) on the basis of their location. The loadings are the coefficients of the original variables that define each principal component [18]. Inertia percentage and correlated variables for axes 1 and 2 are displayed in Table 4. Axes 1 explained 60.85% of the total inertia. Axes 2 explained 24.82% of the inertia and was made positive by arginine, histidine, proline, and aspartic acid. The inertia was made negative by linoleic acid. Plots of the scores in Figure 3 indicated that the data cloud was mainly bidimensional. With respect to the explanatory variables, Figure 4 showed two clusters of varieties. The first cluster included the S and K pod varieties. The second cluster (D and M pod varieties) was individualized. 

## 4. Conclusions

 The fatty acid and amino acid results for okra pods were in agreement with the literature. Quantitatively, total lipids ranged from 4.34 g/100 g to 4.52 g/100 g on a dry weight basis. Okra contents had a significant correlation with the geographical distances. SAT predominated over SFA, and PUFA particularly predominated over MUFA. M pod was found to be the richest in all amino acid values except isoleucine, lysine, and valine. The essential and nonessential amino acids in okra are comparable to those in soybeans. Therefore, okra could be used as a good source of proteins for human nutrition. Seeds can be subjected to oil extraction for additional benefits. Okra can be used in making soaps, emulsifiers, and quick-drying oils and has become increasingly popular in the cosmetics industry.

## Figures and Tables

**Table 1:** Lipid content and geographic location of the four okra samples.

City	Code	Latitude	Longitude	Total Lipids, g/100 g DW
Dakahlia	D	31.053103	31.580615	4.45 ± 0.01^B^
Mansoura	M	31.042536	31.380014	4.34 ± 0.01^C^
Kafr El-Shaikh	K	31.347304	30.80246	4.44 ± 0.02^B^
Suez	S	29.984721	32.524309	4.52 ± 0.02^A^

Values are the average of three individual samples each analyzed in duplicate ±standard deviation. Different uppercase superscript letters, respectively, indicate significant difference (*P* < 0.05) analyzed by Duncan's multiple range test. Contents were determined by Soxhlet apparatus.

**Table 2:** Fatty acid composition (%).

	S	K	M	D
Myristic acid (C14:0)	0.25 ± 0.02^B^	0.49 ± 0.24^A^	0.46 ± 0.10^AB^	0.21 ± 0.04^B^
Pentadecylic acid (C15:0)	0.63 ± 0.01^B^	0.70 ± 0.08^B^	0.70 ± 0.06^A^	0.92 ± 0.07^A^
Palmitic acid (C16:0)	29.18 ± 0.35^B^	43.26 ± 0.11^A^	38.95 ± 2.37^A^	39.51 ± 0.40^A^
Margaric acid (C17:0)	1.44 ± 0.02^C^	1.50 ± 0.06^C^	1.66 ± 0.05^B^	2.16 ± 0.10^A^
Linoleic acid (C18:2)	43.07 ± 0.24^A^	34.40 ± 2.63^B^	33.74 ± 0.95^B^	32.22 ± 0.12^B^
Oleic acid (C18:1)	4.31 ± 0.24^B^	4.55 ± 2.00^AB^	4.47 ± 0.77^AB^	6.98 ± 0.29^A^
Linolenic acid (C18:3)	12.34 ± 0.16^A^	7.82 ± 0.94^C^	10.07 ± 1.06^B^	6.79 ± 0.75^C^
Stearic acid (C18:0)	6.36 ± 0.07^D^	7.28 ± 0.16^B^	6.98 ± 0.23^C^	7.73 ± 0.30^A^
Arachidic acid (C20:0)	2.42 ± 0.04^C^	ND	2.96 ± 0.12^B^	3.48 ± 0.13^A^
Total SAT	40.28 ± 0.33^B^	53.23 ± 0.27^A^	51.71 ± 2.15^A^	54.01 ± 0.95^A^
Total UNSAT	59.72 ± 0.33^A^	46.77 ± 0.27^B^	48.29 ± 2.15^B^	45.99 ± 0.95^B^
Total SAT/Total UNSAT	67.44 ± 0.01^B^	113.81 ± 0.01^A^	107.08 ± 0.10^A^	117.44 ± 0.04^A^
Total PUFA	55.42 ± 0.09^A^	42.22 ± 1.75^B^	43.82 ± 1.67^B^	39.01 ± 0.66^C^
Total MUFA	4.31 ± 0.24^B^	4.55 ± 2.00^AB^	4.47 ± 0.77^AB^	6.98 ± 0.29^A^

Each value is presented as the mean ± standard deviation (*n* = 3). Data with different uppercase superscript letters in the same column of variety respectively indicate significant difference (*P* < 0.05) analyzed by Duncan's multiple range test. ND: Non-detected.

**Table 3:** Amino acid composition (%).

	S	K	M	D
Ile	0.29 ± 0.03^AB^	0.31 ± 0.05^A^	0.29 ± 0.05^AB^	0.22 ± 0.03^B^
Leu	0.70 ± 0.08^AB^	0.72 ± 0.10^AB^	0.78 ± 0.15^A^	0.56 ± 0.04^A^
Lys	0.59 ± 0.07^AB^	0.69 ± 0.09^A^	0.68 ± 0.12^A^	0.50 ± 0.05^B^
Met	0.07 ± 0.02^AB^	0.06 ± 0.02^AB^	0.08 ± 0.02^A^	0.05 ± 0.01^B^
Cys	0.17 ± 0.03^B^	0.17 ± 0.01^B^	0.22 ± 0.03^A^	0.14 ± 0.02^B^

Total sulphuric acids	0.24 ± 0.02^B^	0.23 ± 0.03^B^	0.30 ± 0.05^A^	0.19 ± 0.02^B^

Tyr	0.37 ± 0.04^AB^	0.37 ± 0.05^AB^	0.44 ± 0.08^A^	0.30 ± 0.00^B^
Phe	0.47 ± 0.05^AB^	0.48 ± 0.06^AB^	0.52 ± 0.12^A^	0.36 ± 0.04^B^

Total aromatic amino acids	0.83 ± 0.09^AB^	0.86 ± 0.11^AB^	0.96 ± 0.20^A^	0.66 ± 0.04^B^

Thr	0.45 ± 0.05^A^	0.45 ± 0.06^A^	0.53 ± 0.12^A^	0.38 ± 0.04^A^
Val	0.46 ± 0.05^A^	0.47 ± 0.06^A^	0.45 ± 0.08^A^	0.34 ± 0.04^B^
His	0.24 ± 0.03^B^	0.24 ± 0.03^B^	0.33 ± 0.05^A^	0.23 ± 0.01^B^
Arg	0.67 ± 0.07^C^	0.75 ± 0.09^BC^	1.44 ± 0.17^A^	0.95 ± 0.14^B^

Total essential amino acids (E)	1.82 ± 0.19^B^	1.90 ± 0.25^B^	2.75 ± 0.35^A^	1.89 ± 0.07^B^

Asp	3.23 ± 0.37^B^	2.91 ± 0.27^B^	4.92 ± 1.32^A^	3.58 ± 0.33^AB^
Ser	0.64 ± 0.07^A^	0.61 ± 0.08^A^	0.64 ± 0.18^A^	0.46 ± 0.05^A^
Glu	1.99 ± 0.23^A^	1.82 ± 0.22^A^	2.44 ± 0.86^A^	1.74 ± 0.24^A^
Gly	0.48 ± 0.05^A^	0.49 ± 0.06^A^	0.50 ± 0.11^A^	0.38 ± 0.05^A^
Ala	0.60 ± 0.07^A^	0.61 ± 0.08^A^	0.71 ± 0.19^A^	0.53 ± 0.08^A^
Pro	1.40 ± 0.12^B^	1.38 ± 0.21^B^	2.53 ± 0.77^A^	1.92 ± 0.26^AB^

Total nonessential amino acids (N)	8.34 ± 0.90^AB^	7.81 ± 0.91^B^	11.73 ± 3.42^A^	8.62 ± 0.99^AB^

Total amino acid	12.80 ± 1.37^B^	12.51 ± 1.52^B^	17.49 ± 4.26^A^	12.63 ± 1.07^B^

Each value is presented as the mean ± standard deviation (*n* = 3). Data with different uppercase superscript letters in the same column of variety, respectively, indicate significant difference (*P* < 0.05) analyzed by Duncan's multiple range test.

**Table 4:** Discriminate variables factors of principal components analysis.

	F1	F2
Proper value	15.82	6.45
Variability (%)	60.85	24.82
Cumulative (%)	60.85	85.67
Myristic acid	+3.04	—
Pentadecylic acid	−4.82	—
Palmitic acid	—	+3.01
Margaric acid	—	+5.21
Linoleic acid	—	−8.53
Oleic acid	−5.53	—
Linolenic acid	+2.74	—
Stearic acid	—	+3.66
Arachidic acid	—	+4.79
Ile	+4.70	—
Leu	+6.21	—
Lys	+4.69	—
Met	+5.44	—
Cys	+5.14	—
Tyr	+5.86	—
Phe	+6.24	—
Thr	+5.70	—
Val	+4.99	—
His	—	+7.59
Arg	—	+13.67
Asp	—	+11.68
Ser	+5.84	—
Glu	—	+4.32
Gly	+5.94	—
Ala	+5.35	—
Pro	—	+14.32
